# Guided STED nanoscopy enables super-resolution imaging of blood stage malaria parasites

**DOI:** 10.1038/s41598-019-40718-z

**Published:** 2019-03-18

**Authors:** Jan-Gero Schloetel, Jörn Heine, Alan F. Cowman, Michał Pasternak

**Affiliations:** 1 Abberior Instruments GmbH, 37077 Göttingen, Germany; 2 Walter and Eliza Hall Institute of Medical Research, Parkville, Victoria Australia; 3 Department of Medical Biology, University of Melbourne, Parkville, Victoria Australia

## Abstract

Malaria remains a major burden world-wide, but the disease-causing parasites from the genus *Plasmodium* are difficult to study *in vitro*. Owing to the small size of the parasites, subcellular imaging poses a major challenge and the use of super-resolution techniques has been hindered by the parasites’ sensitivity to light. This is particularly apparent during the blood-stage of the *Plasmodium* life cycle, which presents an important target for drug research. The iron-rich food vacuole of the parasite undergoes disintegration when illuminated with high-power lasers such as those required for high resolution in Stimulated Emission Depletion (STED) microscopy. This causes major damage to the sample precluding the use of this super-resolution technique. Here we present guided STED, a novel adaptive illumination (AI) STED approach, which takes advantage of the highly-reflective nature of the iron deposit in the cell to identify the most light-sensitive parts of the sample. Specifically in these parts, the high-power STED laser is deactivated automatically to prevent local damage. Guided STED nanoscopy finally allows super-resolution imaging of the whole *Plasmodium* life cycle, enabling multicolour imaging of blood-stage malaria parasites with resolutions down to 35 nm without sample destruction.

## Introduction

Malaria remains a global threat, with 216 million cases world-wide and over 400,000 deaths in 2016 alone^1^. There are six parasite species from the genus *Plasmodium* causing malaria in humans: *P. falciparum*, *P. vivax*, *P. ovale curtisi, P. ovale wallikeri, P. malariae* and *P. knowlesi*, with *P. falciparum* being responsible for the vast majority of deaths.

*P. falciparum* undergoes a complicated life cycle which alternates between a female *Anopheles* mosquito and a human host. When the mosquito feeds on human blood, parasitic sporozoites enter the human blood stream and quickly traverse into the liver. There, the parasite undergoes further development and each infected hepatocyte produces up to 40,000 new parasites in the form of merozoites^2^. Merozoites are small, short-lived and highly-specialised for the invasion of red blood cells. They invade erythrocytes in a rapid, multi-step process lasting around 2 minutes^3^. Once the parasite is internalised in the host cell, it is enclosed in a vacuole derived from the host cell plasma membrane and parasite proteins. This structure is called the parasitophorous vacuole and the outside membrane separates the parasite from the host cell cytoplasm (reviewed in^4–6^). Over the next 48 hours, the trophozoite parasite extensively remodels the host cell, establishing pathways that allow nutrient uptake, waste disposal and parasite growth (reviewed in^7^). To achieve this, the parasite exports hundreds of proteins into the host cell^8^. These exported proteins first enter the parasite’s endoplasmic reticulum, where they are trafficked towards the parasite surface and parasitophorous vacuole. The exported proteins are then translocated to the host cell by the recently described protein translocon called PTEX, located on the surface of the parasitophorous vacuole^9–13^. Following translocation, exported proteins end up in the erythrocyte cytoplasm. Several proteins travel further to parasite-derived structures called Maurer’s clefts, which have been suggested to act as protein-sorting organelles^14–16^ and further to the red blood cell surface. The parasite’s proteins displayed on the red blood cell surface, such as the major virulence factor PfEMP1, facilitate cytoadherence of the infected cells to each other and to the blood endothelium^17–21^. This sequesters infected cells from circulation and prevents their destruction in the spleen. During this time, the parasite grows and undergoes cell divisions producing approximately 20 new merozoites^22,23^, which upon cell rapture are released into the blood stream to infect new red blood cells. These cycles of asexual reproduction in the host’s blood are accompanied by the occurrence of malaria symptoms^24^, coincident with diagnosis. Because of this, a major focus for antimalarial drug action is on the killing of blood-stage parasites^25^.

All malaria-causing parasites are minuscule, with merozoites from *P. falciparum* measuring as little as 1.5 μm in diameter. Super-resolution of merozoites^26^ and even liver-stages^27^ of the parasite development has been possible. However, later blood stages pose a major challenge for STED microscopy and imaging beyond the diffraction limit has been restricted to structured-illumination microscopy (SIM, reviewed in^28^). SIM provides only slightly improved resolution (~120 nm) in comparison to diffraction-limited light microscopy (~240 nm), while STED microscopy can reach a resolution of 25 to 35 nm in biological samples^29,30^. In addition, STED has the advantage that it does not require image deconvolution avoiding possible imaging artefacts. More importantly, STED enables highly accurate colocalization studies, as the super-resolved image channels are perfectly aligned by the use of a single STED beam^31^.

The major obstacle in applying STED microscopy in later stages of the parasite life cycle is the presence of so called hemozoin. During its growth, the parasite utilises haemoglobin as its main food source^32^. Since heme, which is released upon haemoglobin digestion, is toxic to the cell, the parasite converts it into an insoluble crystalline form called hemozoin (or malaria pigment), deposited in the food vacuole^33,34^. Interfering with the formation of the hemozoin has been a major target for antimalarial drugs^35–37^. However, hemozoin poses a major challenge during the imaging of malaria parasites. It is highly reflective, making it very difficult to study by confocal microscopy, and it partly absorbs energy from high-power lasers, such as those used in STED. The resulting heat has been well characterised through photoacoustic spectroscopy^38–41^ and leads to hemozoin or β-hematin disintegration and sample destruction^38–41^. Photodamage is expected for most STED lasers, including the commonly used 592 nm, 595 nm, 660 nm, 775 nm and 810 nm lasers, as hemozoin absorbs^38,42–44^, scatters^45^ and converts light to heat in this spectral range^38,39,41,42^. Energy absorption tends to decrease slightly with wavelength, but heating is still evident at 700 nm^42^ and 820 nm^39^ and particularly severe around 670 nm^41^.

A recently obtained mutant of *P. berghei* with increased resistance to chloroquine may promise a biological strategy to limit sample damage, as it shows reduced formation of hemozoin^46^. Although the parasites were viable upon infection of reticulocytes, they had a reduced size, produced fewer merozoites and were unable to complete their growth in mature erythrocytes. However, as reticulocytes are very short-lived and represent only a small proportion of red blood cells (reviewed in^47^), the majority of studies are conducted on infected erythrocytes, where hemozoin poses a much greater challenge.

Adaptive illumination (AI) STED methods have been developed recently to reduce photodamage to sensitive samples. The two main AI STED approaches, REduction of State transition Cycles (RESCue) STED^48^ and Dynamic Intensity Minimum (DyMIN) STED^30^, adapt illumination based on the fluorescence signal and reduce total light exposure 20-fold compared to typical STED methods. While these approaches reduce photobleaching, they do not prevent the destruction of samples containing hemozoin, as high laser intensities still occur locally irrespective of the presence of hemozoin. Here we describe guided STED, a novel AI STED approach that not only allows super-resolution imaging of hemozoin-rich samples but also enables visualisation of the hemozoin itself with unprecedented degree of detail.

## Methods

### Parasite culture and sample preparation

Human malaria parasites *P. falciparum* line CS2 were maintained as asexual stages in erythrocytes (blood group O, 4% haematocrit, Australian Red Cross) in RPMI-HEPES medium supplemented with 0.2% sodium bicarbonate (Sigma), 5% heat-inactivated human serum and 0.25% Albumax (ThermoFisher) as described previously^49^. To obtain synchronised late-stage parasite cultures, parasites were synchronized using 5% sorbitol (Sigma) for 8 min and cultured for 24 hours^50^. All parasites were cultured in sealed incubators in an atmosphere consisting of 5% CO_2_, 1% O_2_ and 94% N_2_.

### Immunofluorescence

Sample fixation was undertaken either on blood smears with ice-cold mixture acetone:methanol (9:1) for 10 min; or in suspension in a solution of 4% paraformaldehyde with 0.0075% glutaraldehyde in PBS for 30 min at room temperature, followed by 20 min permeabilisation using 0.1% Triton-X 100 as described previously^50,51^: All samples were blocked in 2% bovine serum albumin in PBS at room temperature for 1 hour, followed by an incubation with primary antibodies at 4 °C (overnight for PFA-fixed samples or 2 hours for acetone -fixed samples). Primary antibodies were used at the following dilutions in the blocking solution: rabbit anti-EXP2 1:500^11,13^, mouse anti-EXP2 1:500^11,52^, rat anti-HA 1:100 (Roche, Clone 3F10), rabbit anti-ERC 1:1000^53^. Samples were then incubated in secondary antiobodies diluted in blocking solution at 1:1000 dilution (Abberrior STAR 635P or 580) Following three further washes with PBS, samples were settled onto polyethyleneimine-coated coverslips (Sigma) and mounted in mowiol (Sigma) or ProLong^TM^ Diamond Antifade (ThermoFisher Scientific). Nuclear staining was performed using SiR DNA kit (Spirochrome) according to manufacturer’s instructions.

### Bead samples

Coverslips (22 × 22 mm, thickness #1.5, Marienfeld) were coated with poly-L-lysine (Sigma) for 10 min at room temperature before incubation with a mixture of gold nanoparticles (80 nm diameter, BBI solutions) and fluorescent TetraSpeck^TM^ beads (100 nm diameter, ThermoFisher Scientific) in PBS buffer. After 15 min, excess liquid was removed and the coverslip was mounted on glass microscopy slides (VWR) with DPX mountant (Sigma).

### Optical setup

The optical setup described previously^30^ was modified to allow guided STED with malaria samples (see Supplementary Fig. 1). To create a separate light path for reflected light detection, a 10:90 (R:T) non-polarising beam-splitter cube (Thorlabs) was introduced into the detection light path behind the long pass filter that inserts the custom-build STED laser (wavelength 775 nm, pulsewidth ~500 ps, maximum power 3.5 W). Even though the long-pass filter removes the majority of the reflected STED laser light, it was attenuated further by several neutral density filters adding up to 3.3–4.6 OD to avoid detector saturation. For some images, the neutral density filters were followed by a dichroic mirror (F73-078 STED beam-splitter ZT 780, AHF Analysentechnik) to specifically attenuate reflected light of the STED laser. The intensity ratio of reflected STED laser light and excitation laser light was adjusted by changing the angle of the dichroic mirror. The parallel reflected light was then focussed on the multimode fibre of a fibre-coupled avalanche photodiode detector (Single Photon Counting Module, SPCM-AQRH-13-FC, Excelitas Technologies) using an achromatic doublet lenses (f = 30, Thorlabs). The lens combination in combination with the fibre diameter creates a ~3.5 AU pinhole.

### Conventional and guided STED imaging

STED measurements were performed on a modified commercial easy3D STED microscope (2C QUAD Scan easy3D Super-Resolution microscope, Abberior Instruments) with avalanche photodiodes (Single Photon Counting Module, SPCM-AQRH-13-FC, Excelitas Technologies) for detection.

Confocal fluorescence images of Abberior STAR 635P and Silicon Rhodamine stainings were recorded using a pulsed 640 nm excitation laser (Abberior Instruments) and detection at 650–720 nm. Abberior STAR 580 was excited with a pulsed 561 nm laser (Abberior Instruments) and detected at 580–630 nm. Hemozoin was imaged detecting reflected light from either the pulsed 775 nm STED laser running at low power (0.49–2.9 mW measured in the sample plane) or from the 640 nm or 561 nm excitation lasers. The same excitation lasers and detection range were used for STED imaging, with added time-gating of the fluorescence signal.

Guided STED imaging was performed using 1–3 probe steps to reduce damage to hemozoin and the sample. All guided STED versions included a hemozoin probe step detecting reflected light of the STED laser running at low power (0.49–2.9 mW). Optionally, a hemozoin probe step detecting reflected light of the 640 nm or 561 nm excitation laser was added for increased accuracy of hemozoin detection. For each hemozoin probe step, the user defined a signal threshold above which further illumination of the pixel with the STED laser at high power was prevented. Another optional probe step was a standard confocal step detecting fluorescence of the respective dye with a threshold below which further illumination of the pixel with the STED laser was prevented.

Two-colour STED and guided STED images were recorded frame sequentially for each dye. Reflective control images of hemozoin were recorded after (guided) STED images of each dye and added up for the final image. To compare damage caused by the STED laser (Fig. 3), STED imaging was performed with the STED laser active but excitation lasers inactive in most frames to reduce photobleaching.

## Results

### Guided STED development

**Figure 1:**
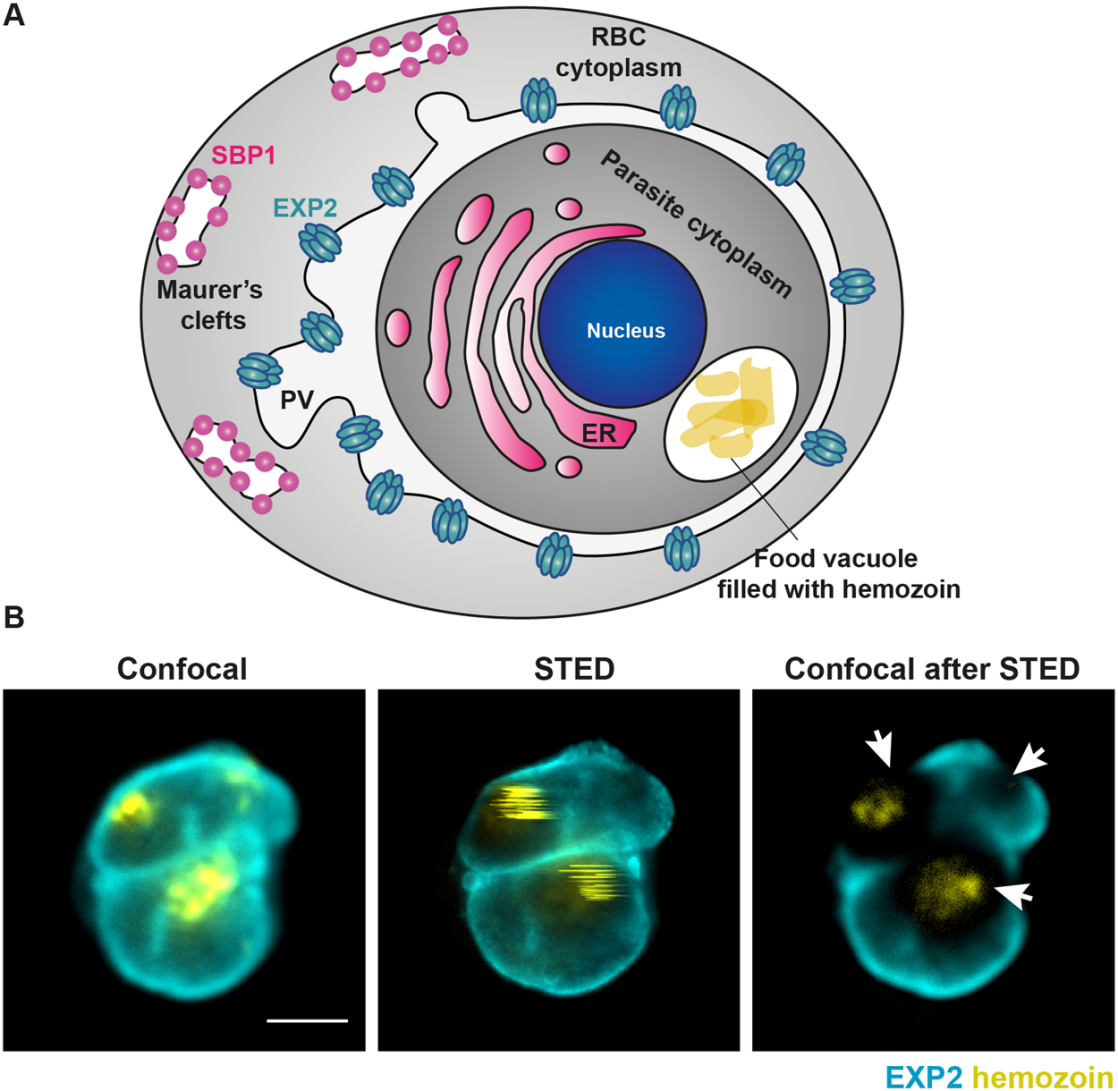
The hemozoin challenge in malaria samples. (**A**) Schematic representation of a red blood cell infected with human malaria parasite *Plasmodium falciparum* at a mid- trophozoite stage. The position of hemozoin-rich food vacuole has been marked as well as several parasite-derived structures important for parasite protein export into the host cell. ER – endoplasmic reticulum, PV – parasitophorous vacuole surrounding the parasite, RBC – red blood cell, EXP2 – the core component of parasite protein translocon, SBP1 – a marker of Maurer’s clefts which are parasite-derived structures that facilitate protein trafficking to the surface of infected red blood cell. **(B)** Confocal and conventional STED images image of an infected red blood cell stained for EXP2 marking parasitophorous vacuole (cyan) and hemozoin in the food vacuole visualised via reflected light of the STED laser (yellow). Conventional STED increases the resolution but damages hemozoin and any surrounding cellular structures as evidenced in confocal images recorded after a single frame of STED imaging. Scale bar 2 μm.

In order to obtain super-resolution images of late blood stages of malaria-causing parasites, we needed to develop an approach that would allow us to overcome the challenges posed by the presence of hemozoin. Major sample destruction was particularly prominent in the regions adjacent to the parasite food vacuole while it was still possible to obtain super-resolution images of parasite surface (Fig. 1). This precludes imaging of parasite organelles in close proximity to hemozin crystals, such as the food vacuole or the endoplasmic reticulum. We therefore reasoned that we could obtain super-resolution images of close to the entire parasite only if we could avoid irradiating the hemozoin with high-power STED laser. To achieve this, we took advantage of a recently developed AI STED approach, termed RESCue STED^48^. RESCue reduces the light dose received by the sample during STED imaging by switching off the high-power STED laser in sample areas without fluorescent structures. While no super-resolution information can be gained from non-fluorescent areas, unnecessary activation of the STED laser here still damages neighbouring fluorescent structures owing to the large size of the donut-shaped point spread function (PSF) of the STED laser. Non-fluorescent areas are defined during the scan if the fluorescence signal in standard confocal mode drops below a user-defined value. This evaluation, called “probe step” is completed within the pixel dwell time (microseconds) using fast electronics. Only if appropriate, the pixel is immediately illuminated with the STED laser for super-resolution during the remaining pixel dwell time. However, this evaluation is made irrespective of the presence of hemozoin, permitting strong illumination of hemozoin particles resulting in sample damage. Therefore, the original RESCue approach can provide only limited protection for samples containing hemozoin, and only if the fluorescent structures are not located near the hemozoin particles.

**Figure 2:**
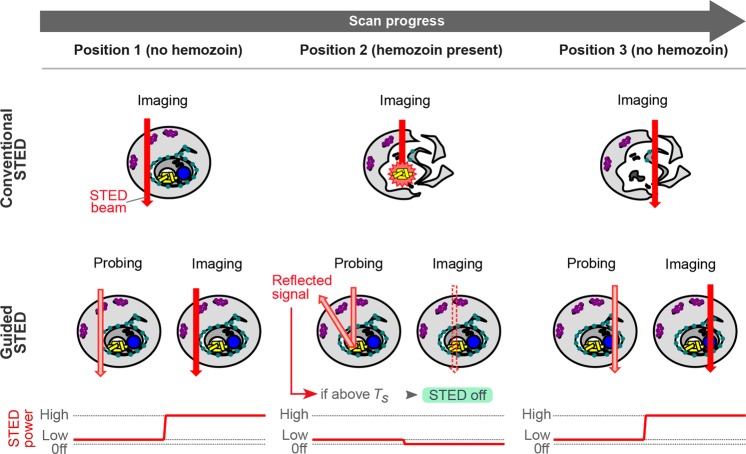
Principle of guided STED for malaria samples. In conventional STED super-resolution microscopy (top row), the infected red blood cell is scanned with the STED laser (red arrow) at full power in every pixel leading to disintegration of hemozoin particles (yellow) and sample destruction. Guided STED (bottom row) can test for the presence of hemozoin at every pixel using light reflected by hemozoin particles (probing). To avoid sample damage during the probing step, the depletion laser is running at a very low power (pink). Only where the reflected light signal is below the threshold T_S_, the depletion laser is switched back to high power (red arrow) for super-resolution imaging.

Adaptation of the AI STED approach for imaging of *P. falciparum* first required a means of detecting sensitive hemozoin structures to avoid their disintegration and resulting sample destruction. Transmitted light detection resulted in strong, ring-shaped interference patterns precluding safe identification of hemozoin particles. Therefore, we took advantage of the highly reflective nature of hemozoin particles for direct imaging of hemozoin via detection of reflected light of the excitation laser (Fig. 2). The reflected light signal was several orders of magnitude stronger than fluorescence signals. This allowed a detailed visualisation of hemozoin but required signal dampening using neutral density filters. In turn, a positive hemozoin detection signal would result in switching off the STED laser whenever a defined signal threshold was exceeded. Thanks to the relatively low power, excitation lasers could be safely employed to detect hemozoin without any sample damage. While the interplay of excitation and STED lasers is important for fluorophore bleaching^54^, for hemozoin damage multiple laser pulses show additive effects on sample heating^40^ indicating a negligible role played by the excitation lasers here. Unfortunately, the excitation laser PSF is inherently much smaller than the PSF of the STED laser. Therefore, the excitation lasers would not cover all of the area illuminated by the larger STED beam (Fig. 2). For this reason, the excitation laser was not sufficient to define the areas requiring deactivation of the STED beam.

On the other hand, the STED laser itself has the right shape but is far too powerful to be used for a non-destructive probe step. This was only overcome with the aid of DyMIN STED^30^, a recent improvement of the AI STED approach. DyMIN employs new electronics and software to allow not only simple blanking of the STED and excitation lasers, but multilevel variation of the STED laser power to record additional probe steps at intermediate resolution. Here, we were able to utilise the STED doughnut at low intensity (0.49–2.9 mW in the sample plane) for a probe step with non-destructive detection of hemozoin via reflected light. If no hemozoin was detected during the probe step, the STED power was immediately raised to 24–71 mW for super-resolution imaging.

Finally, to complete the requirements for STED imaging with parasite samples, the detection of reflected light was optimised. To overcome the long rise-time and signal variations of the commonly used photo-multiplier tubes, we switched to an avalanche photodiode detector (APD). A separate detection beam path with a larger effective pinhole size was employed to allow detection of the complete STED doughnut PSF.

### Guided STED allows imaging close to hemozoin without sample perturbation

We then sought to test the advantages of guided STED by comparing the damage caused to the sample while imaging using the new guided STED approach, conventional STED imaging or STED imaging with simple breaks added in between pixel illumination steps for a relaxation of the energy absorbed by hemozoin. In an imaging series, the power of the STED laser was gradually increased and after each exposure the survival of hemozoin and cellular structures was checked using the reflective light signal and also standard confocal fluorescence imaging. As we were interested only in the induced damage, STED imaging was performed mostly without excitation of the fluorophores to reduce bleaching and thus enable a complete imaging series.

**Figure 3:**
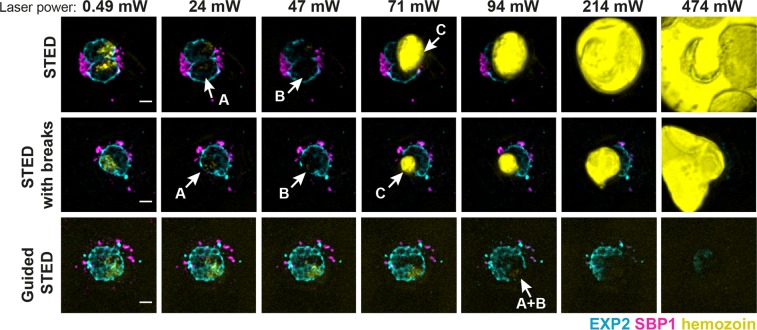
Sample damage in conventional or guided STED. Representative confocal images of infected red blood cells stained for the parasitophorous vacuole (EXP2, cyan) and Maurer’s clefts (SBP1, magenta) and hemozoin visualisation (yellow) using reflected light from the excitation laser. Images were recorded after single rounds of STED imaging with increasing powers (0.49, 24, 47, 71, 91, 214 or 474 mW) of the STED laser to compare damage inflicted in conventional STED mode, conventional STED mode with illumination breaks and guided STED mode. Examples of three major stages of sample damage: **A** – altered hemozoin structure, **B** – damages of fluorophores in the proximity of hemozoin, **C** – hemozoin disintegration. Scale bar 2 μm.

We identified three major steps of sample damage caused by the STED laser. Firstly, we observed morphological changes in the hemozoin as evident from the altered light reflection in the hemozoin area (at about 24 mW laser power, Fig. 3A). Secondly, a further increase of the laser power led to an excessive damage of fluorophores in the proximity of the hemozoin (at 36 to 47 mW laser power, Fig. 3B) after a single scan. Thirdly, laser powers greater than 47 mW resulted in a complete disintegration of the hemozoin and destruction of the entire infected red blood cell (Fig. 3, yellow, Supplementary Movie 1) with mechanical damage to the sample^41^ and hemozoin products spreading through the mounting medium. We then tested whether introducing simple short breaks (120 µs) with inactive lasers between the STED imaging pixel steps (30 µs) to allow energy dissipation could reduce the damage inflicted to the sample. This approach led to only minimal improvement in sample survival. Therefore, we were able to exclude the possibility that any improvement seen with guided STED would be due to the low-power probe step acting as a break between pixels. In conclusion, imaging of the sample with conventional STED was only possible when using less than 24 mW laser power, regardless of whether the recovery breaks were introduced (Fig. 3). Such a low value precludes obtaining high-quality STED images from biological samples, which commonly need laser power significantly greater than 24 mW.

In contrast, guided STED caused no apparent sample damage with laser powers up to 71 mW (Fig. 3). First stages of sample damage, i.e. fluorophore bleaching, were observed only when the STED laser power exceeded 94 mW. The complete disintegration of hemozoin was never observed indicating that the hemozoin particles remained intact, as confirmed by the reflected light image (Fig. 3).

### Imaging of parasite subcellular structures using guided STED

**Figure 4:**
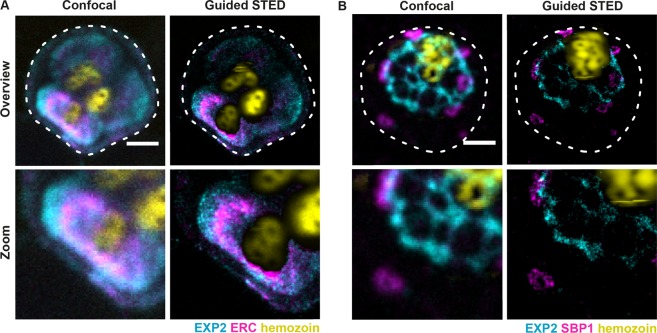
Two-colour imaging of parasite structures using guided STED. Confocal and guided STED images of infected red blood cells. Staining for EXP2 (cyan) and ERC (magenta, **A**) or for EXP2 (cyan) and SBP1 (magenta, **B**). Lower panels show a magnified view of respective structures at super-resolution. The ER (magenta, **A**) and parasitophorous vacuole (cyan, **A** and **B**) are located close to the hemozoin particles (yellow) which are visualised via reflected light of the STED laser. Guided STED prevented sample damage by switching off the STED laser in areas with high amounts of hemozoin. Scale bar 2 μm.

To validate our approach, we used guided STED to image several *P. falciparum* organelles that occupy distinct cellular regions and that are critical for the parasite growth inside the host cell. We imaged the parasite endoplasmic reticulum, which is the first destination for the parasite proteins directed for export into the host cell^55^ and which occupies space in very close proximity to the food vacuole and the hemozoin, thus providing a major challenge for STED imaging. To visualise the parasite endoplasmic reticulum, we used antibodies against ERC, which has been shown to localise to the ER^56^ (Fig. 4A). We also labelled EXP2, the core component of the parasite protein translocon or PTEX, which provides the nexus for the entry of parasite proteins into the infected red blood cell and is essential for the survival of the parasite in the host cell^9,10,12,13,57,58^. EXP2 labels the parasitophorous vacuole which separates the parasite from the host cell (Fig. 4A,B). Finally, we also visualised Maurer’s clefts, parasite derived organelles which reside inside the infected red blood cell and facilitate protein trafficking to the erythrocyte surface^14,15^. For this, we used SBP1 as a known marker of Maurer’s clefts (Fig. 4B)^59^. We used these probes to obtain 2-colour guided STED images of either EXP2 and ERC (Fig. 4A) or EXP2 and SBP1 (Fig. 4B). In addition, we also visualised the hemozoin particle using reflected light (Fig. 4A,B). Using guided STED, we obtained first STED images of late blood-stage malaria parasites. We achieved an unprecedented resolution of 35 nm (Supplementary Fig. 2) with the hemozoin particle remaining intact. This was despite imaging as close as 300–400 nm to the hemozoin particles (Supplementary Fig. 3) permitting successful two-colour STED images (Fig. 4).

### Further improvements to guided STED for malaria samples

Initial results using guided STED met our requirements. However, on some occasions, slight interference patterns in the reflected light image of the STED beam caused hemozoin to go undetected in a few individual pixels, e.g. at the centre of the STED laser donut. Depending on the STED laser power, this could potentially be enough to trigger the disintegration of the hemozoin. To add a second safety feature, the reflected light of an excitation laser with a standard gaussian PSF was harnessed in an additional probing step to ensure complete detection of hemozoin. To this end, the reflected light of the much stronger STED laser was specifically dampened by a dichroic mirror before general dampening of all reflected light to avoid detector saturation (Supplementary Fig. 1). In summary, this allowed us to perform two sequential probe steps before switching the STED laser to high power or off: A first step detecting reflection of the STED laser beam and a second step detecting the reflection of the excitation laser beam. Introducing two probe steps allowed for more reliable hemozoin detection and a larger error margin when adjusting the detection thresholds, as hemozoin signals from both probe steps are partially overlapping and partially complementing each other in fringe areas. This allowed reliable two-colour guided STED imaging with settings, which, in conventional STED mode, would consistently result in complete sample destruction, even when recording only one frame with one colour (Supplementary Fig. 4).

We then achieved further improvements in the imaging of hemozoin-containing cells by combining guided STED for *P. falciparum* samples with the original RESCue approach^48^. Here, the STED beam was not only switched off in the presence of hemozoin, but it was only activated in the presence of fluorescent structures. For this purpose, one or two guided STED probe steps using reflected light are followed by an original RESCue probe step that identifies fluorescent structures in standard fluorescence mode. In order to detect reflected STED light and reflected excitation laser light within one image acquisition, both channels had to be attenuated to comparable levels. A combination of all three probe steps enabled super-resolution volume imaging of *P. falciparum*-infected erythrocytes containing hemozoin (Supplementary Movie 2).

The general approach of combining guided STED with the original RESCue approach proved very efficient and could be demonstrated in a model system consisting of fluorescent beads mixed with reflective gold nanoparticles (Supplementary Movie 3). Similar to hemozoin, gold nanoparticles disintegrated when irradiated with the STED laser at high intensity. Using all three probe steps, we were able to record super-resolution movies at STED laser powers up to 119 mW (Supplementary Movie 3D,H). Three masks visualising pixels where the STED beam was deactivated (Supplementary Movie 3, bottom row) due to decisions made in the three probe steps, illustrate how the individual probe steps complement each other for best protection. The detection gap at the centre of the STED beam (Supplementary Movie 3F,G, cyan area) was easily filled by hemozoin detection using the excitation laser (Supplementary Movie 3E–H, yellow area). Switching the STED laser off due to lack of fluorescent structures (Supplementary Movie 3E–H, magenta areas) provided additional protection for particles without neighbouring or overlapping fluorescent structures (also used in Supplementary Fig. 3). All three probe steps could be combined freely to suit different samples, depending on the required STED laser power and the density of fluorescent labels in the sample.

## Discussion

Development of super-resolution techniques opened up new opportunities in research on small intracellular parasites^28^. However, the use of super-resolution microscopy in the studies of malaria-causing parasites has been limited to early stages of the parasite life cycle^26^ or to techniques that provide only moderate improvement in the resolution, such as SIM^50^. In particular, STED microscopy has not been possible on late blood-stage malaria parasites due to the presence of hemozoin and the sample’s susceptibility to light, precluding imaging of key processes essential for parasite survival, such as protein trafficking and hemozoin formation that occur at later stages of red blood cell infection. This is particularly important in the context of the export of parasite proteins into the host cell as they travel between multiple parasite-derived organelles that are very small in size^7,60^. Recent identification and structural resolution of the parasite trafficking machinery, in particular the PTEX translocon complex^9,13,61,62^ has created an increased demand on high-resolution imaging techniques^11,50^. Furthermore, both parasite protein export^63^ and hemozoin formation^64^ have been shown to provide validated targets for the development of antimalarial drugs^25^. In fact, artemisinin, chloroquine and mefloquine, each major antimalarial drugs, all target aspects of hemozoin formation^64,65^. Our guided STED approach for malaria samples can not only help to understand the processes occurring in the parasite when hemozoin is present in the cell, but the visualisation of the reflected light used in our approach can provide a useful tool to study hemozoin formation itself and aid in studies on drugs interfering with this process. We believe that guided STED will provide a major tool for the studies on *Plasmodium spp*. and can be applied in studies on other blood parasites that produce hemozoin particles, such as *Trypanosoma* and *Schistosoma*^37,66^.

The guided STED approach may also be extended to benefit different types of sensitive samples, for example highly pigmented cells and plants. So far, plant samples have been underrepresented among STED super-resolution studies^67,68^ as chlorophyll and natural dyes strongly absorb STED laser light, limiting STED imaging mostly to the root^69^ or ligneous parts^70^. Here, guided STED may protect those structures most enriched in chlorophyll, which is easily detected using autofluorescence. Our successful imaging of gold particles suggests that guided STED could also be applied in correlative super-resolution light-electron microscopy, as gold beads are often used for image registration (reviewed in^71,72^).

While the original RESCue and DyMIN STED approaches offered greatly reduced bleaching of fluorophores, guided STED adds a fundamentally new functionality to the AI STED principle, preventing sample damage by avoiding sensitive regions. The gain of accessing difficult samples is only slightly limited by the fact, that the most sensitive structures are excluded from super-resolution imaging. Effectively, STED super-resolution microscopy benefits quantitatively from AI STED, increasing resolution, brightness or number of possible frames, and qualitatively from guided STED by unlocking new sample types. Enabling STED brings a range of unrivalled super-resolution capabilities to these samples, including fast and direct acquisition at highest resolution, perfectly aligned image channels for colocalisation studies^31^ and the option to combine super-resolution with methods such as FLIM^31,73^, FCS^74^ and two-photon *in-vivo* imaging^75^.

In addition to preventing damage to fixed samples during STED microscopy, in the future guided STED should be well suited for live cell imaging. The pixel-wise (µs range) or line-wise (ms range) switching processes are faster than many observable cellular movements, thus preventing motion blur or laser switching errors. The major challenge, however, is posed by the viability of the most light-sensitive samples. Processes such as parasite egress or merozoite invasion can be particularly susceptible to light (unpublished data), requiring assessment of sample viability on a case to case basis. Even standard confocal microscopy can affect live organisms, often requiring measures such as alternative imaging techniques, as reported for highly sensitive organisms such as corals^76^, suboptimal illumination with sacrifices in raw image quality and a need for image restoration^77^, or even modification of the model organisms, such as zebra fish larvae, to reduce the strongly absorbing pigmentation^78^. As long as the most sensitive structures are known and can be identified by fluorescent, reflective or opaque markers, a guided STED or guided confocal approach may offer a chance to image with greater brightness and higher resolution while avoiding extensive illumination of the most sensitive regions.

## Supplementary information

Supplementary movie 1: hemozoin challenge

Supplementary movie 2: Volume imaging using guided STED

Supplementary movie 3: Super-resolution movies in a bead model system

Supplementary Information
