# Supplementary material for: Guided STED nanoscopy enables super-resolution imaging of blood stage malaria parasites
Source: Sci Rep. 2019 Mar 18;9:4674. doi: 10.1038/s41598-019-40718-z (PMC6423018; doi:10.1038/s41598-019-40718-z)
Supplement: Supplementary file 4 — Supplementary Information [file 41598_2019_40718_MOESM4_ESM.pdf]

Title: Guided STED nanoscopy enables super-resolution imaging of blood stage malaria parasites

Jan-Gero Schloetel[1]*, Jörn Heine[1], Alan F. Cowman[2,3], Michał Pasternak[2,3]*

[1]Abberior Instruments GmbH, 37077 Göttingen, Germany
[2] Walter and Eliza Hall Institute of Medical Research, Parkville, Australia
[3]Department of Medical Biology, University of Melbourne, Parkville, Victoria, Australia
*authors contributed equally
Correspondence: pasternak.m@wehi.edu.au (M.P.) and g.schloetel@abberior-instruments.com (JG.S)

Supplementary Information

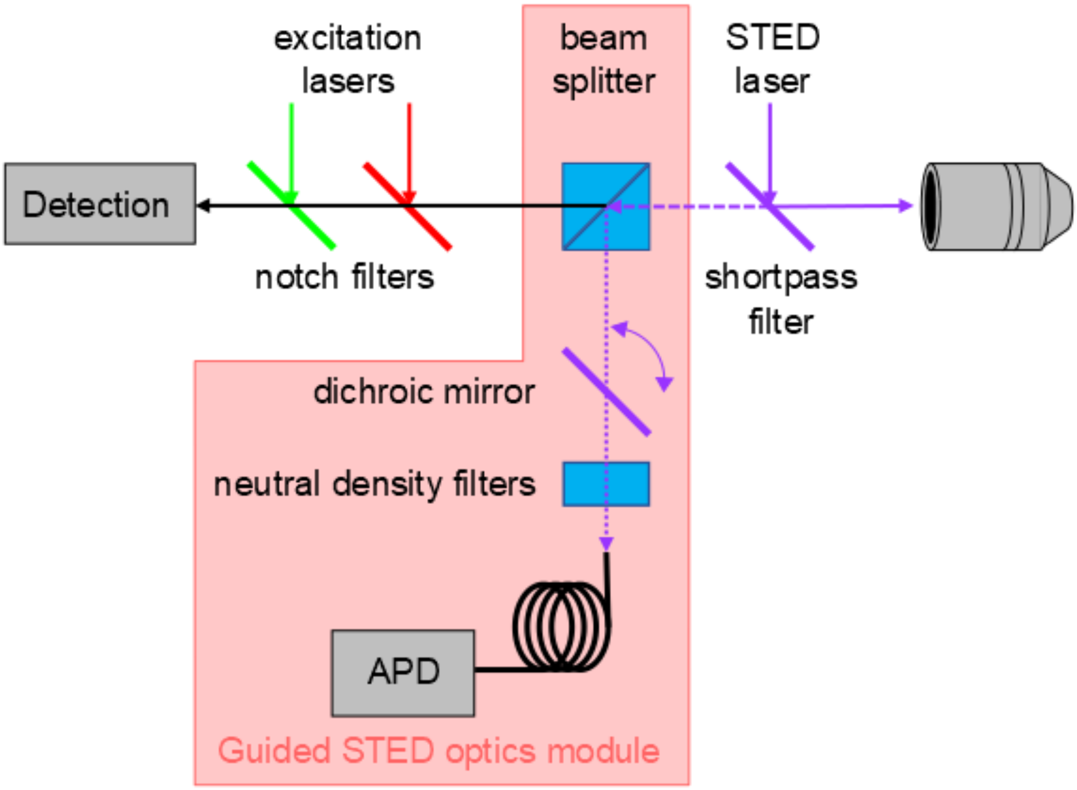

Supplementary figure 1: Optical setup
Excitation lasers (561 nm and 640 nm) were introduced to the beam path using notch filters and 90% of their light passed a 10:90 (R:T) non-polarising beam-splitter cube before entering the scanner unit towards the objective. The STED laser (775 nm) was introduced to the beam path using a short pass filter without passing through the beam splitter to avoid polarisation

changes completely. Reflected light returning from the sample first passed the short pass filter, removing a large proportion of the reflected depletion laser light. The beam splitter cube separated 10% of the remaining reflected light which was then passed through neutral density filters for further attenuation. Optionally, an additional dichroic mirror to was introduced for specific attenuation of the reflected depletion laser light. The attenuated reflected light was then focussed onto the fiber of a fiber-coupled avalanche photodiode detector (APD). Fluorescence light coming from the sample passed through the short pass and notch filters with a loss of 10% caused by the beam splitter cube.

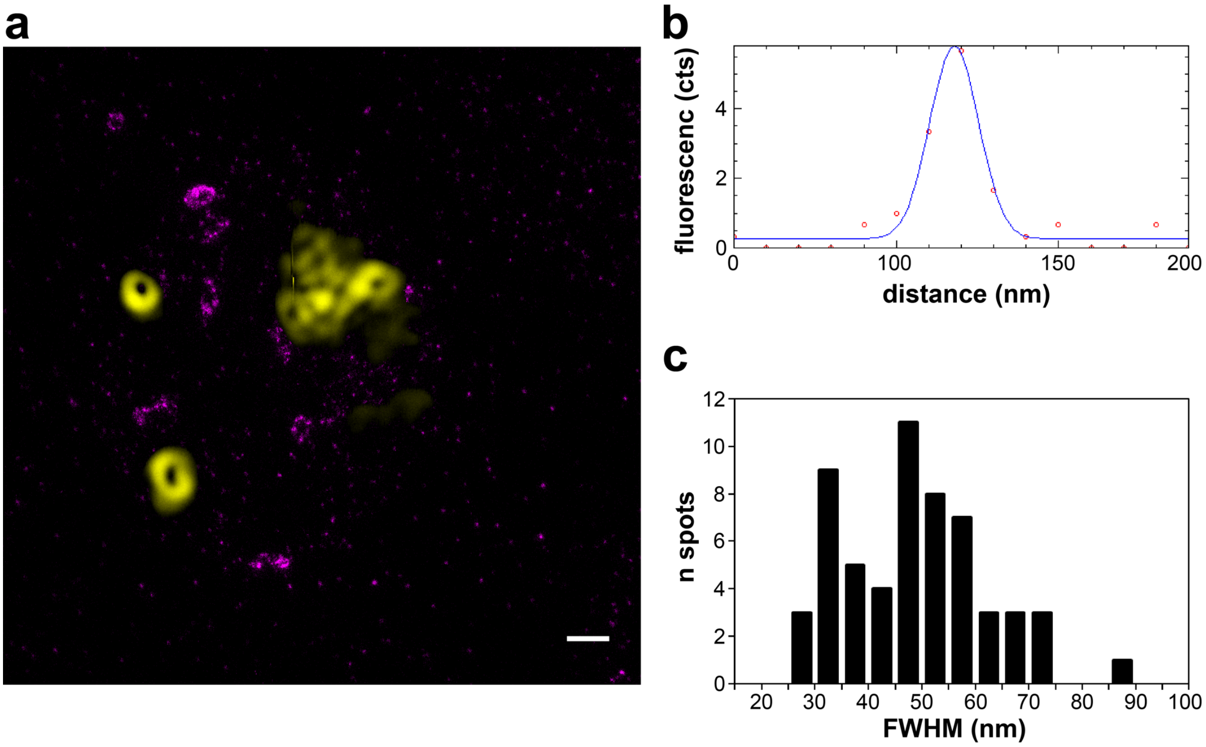

Supplementary figure 2: Resolution measurement

A guided STED image (a) of an infected red blood cell stained for SBP1 (magenta, marker of Maurer's clefts) and containing hemozoin (yellow) was recorded with a pixel size of 20 nm (scalebar 1 µm). Small spots outside the main Maurer's cleft structures, potentially originating from single or few primary antibodies, were treated as point sources for size measurements. Intensity profiles (b, red) were measured in the indicated regions of interest, averaging over a width of three pixels, and fitted with a Gaussian function (b, blue). A histogram (c) shows the full width at half maximum (FWHM) of 57 spots after removal of fits with an R2 below 0.9 and visual inspection.

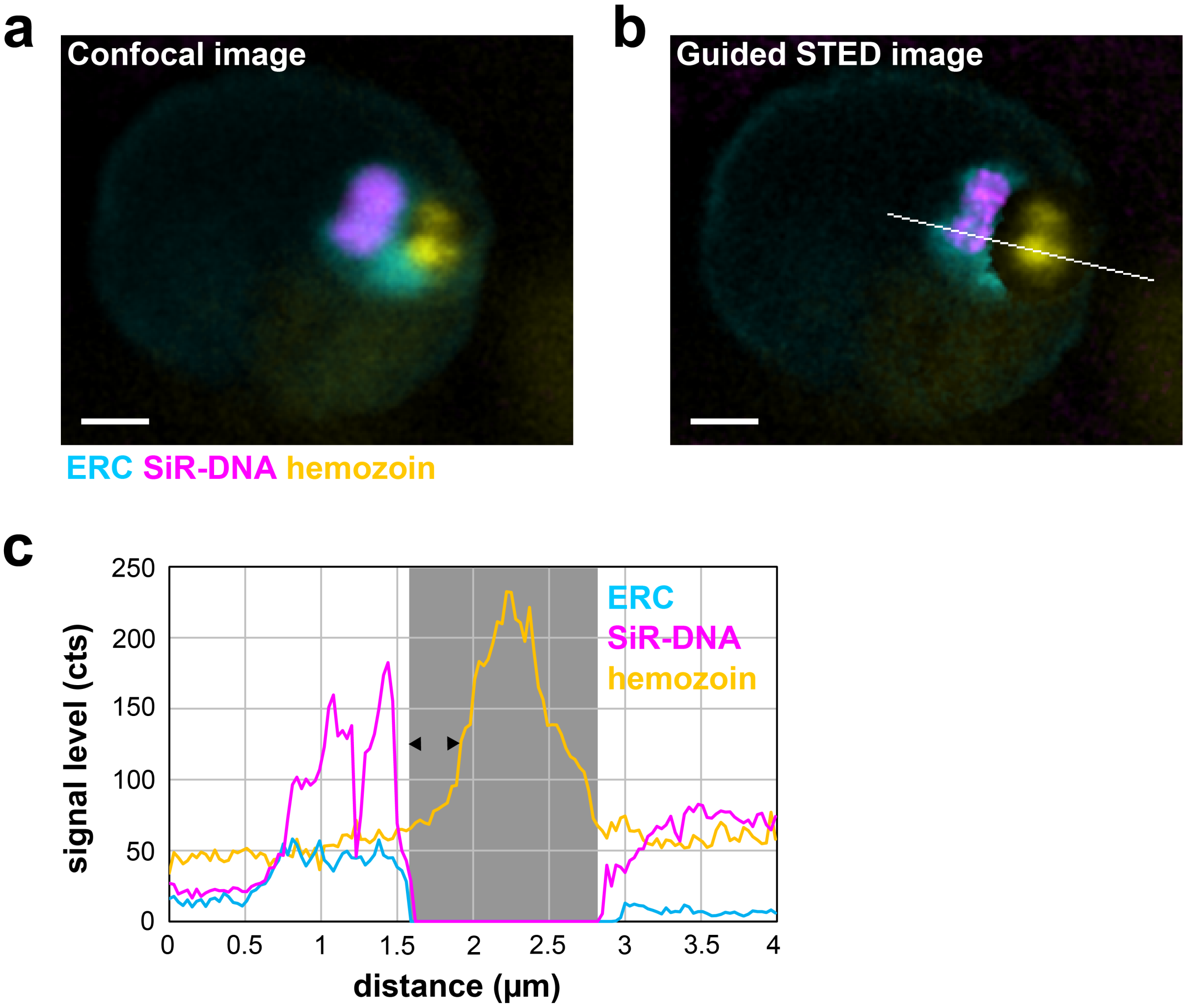

Supplementary figure 3: Imaging exclusion zone around hemozoin particles
Confocal image of an infected red blood cell (A) stained for parasitic ERC (cyan, marker of the ER) and DNA (magenta) with hemozoin (yellow) visualisation via reflected light of the 640 nm excitation laser (scalebar 1 µm). Using guided STED (B), an area around the hemozoin particles was excluded from STED imaging to avoid sample damage. Measuring along a line (B, white) in the guided STED image, the extent of the exclusion zone was determined using intensity profiles (C). The exclusion zone edge (grey) ended around 300 nm away from the from the hemozoin particle image (see black arrow), not accounting for the width of the excitation laser spot (~200 nm). Images were smoothed using a Gaussian filter.

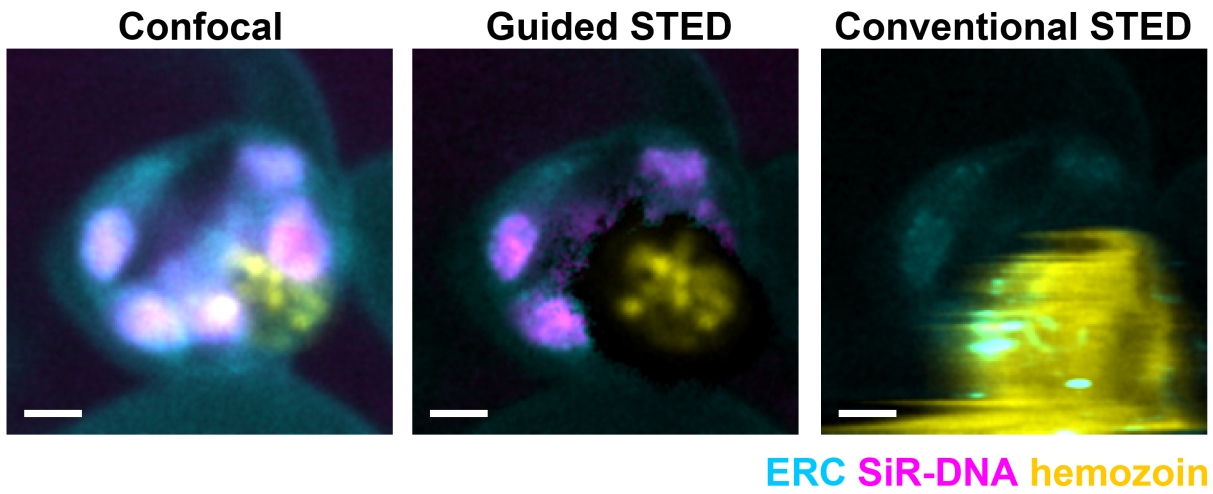

Supplementary figure 4: Guided STED with two reflected light probe steps

Infected red blood cells with parasites at the trophozyte stage were stained for ERC (cyan) and DNA (magenta). Super-resolution images were recorded using guided STED with two reflected light probe steps, one detecting hemozoin via reflected light of the 640 nm excitation laser (yellow) and another detecting reflected light of the STED laser. For comparison, confocal channels were recorded simultaneously. The combination of two probe steps provided robust protection of the sample for frame-sequential dual colour super-resolution imaging. Repeating the same super-resolution acquisition of just one colour channel in conventional STED mode resulted in complete sample destruction. Images were smoothed using a Gaussian filter. Confocal and guided STED images were recorded simultaneously. Scale bar 1 µm.

Supplementary movie 1: hemozoin_challenge
Representative confocal images of infected red blood cells stained for the parasitophorous vacuole (EXP2, cyan) and Maurer's clefts (SBP1, magenta) and hemozoin imaged using reflected light from the excitation laser. Images were recorded after single rounds of STED imaging with increasing powers (100% equals 474 mW) of the depletion laser. Scale bar 2 μm.

Supplementary movie 2: Volume imaging using guided STED
Animated Z-stack of an infected red blood cell stained for ERC (magenta) with hemozoin (yellow) visualisation via reflected light of the 640 nm excitation laser. For comparison, images were recorded in standard confocal mode and in guided STED mode with a step size of 100 nm along the Z-axis. Scalebar 1 μm. SiR-DNA stain was present but not imaged.

Supplementary movie 3: Super-resolution movies in a bead model system
A mixture of gold nanoparticles and fluorescent beads was imaged with depletion laser powers of 14 mW (**A/E**), 47 mW (**B/F**), 71 mW (**C/G**) and 119 mW (**D/H**). As the resolution of the fluorescent bead image (magenta, top row) increased, the pixel size was decreased from 30 nm (**A/E/B/F**) to 20 nm (**C/G**) and 15 nm (**D/H**), further increasing the total illumination of the sample.
In guided STED mode, the gold nanoparticles were protected from strong depletion laser light employing three probe steps: Reflected light of the excitation laser rendered an accurate image of the gold bead (top row, yellow) and triggered the decision to switch the depletion laser off (bottom row, yellow area). Similarly, reflected light of the depletion beam at low-power triggered switch-off (bottom row, cyan area, partially overlapping yellow area). Finally, areas without fluorescent beads (pink) were identified in standard confocal imaging mode to switch off the depletion laser. After 30 frames, STED imaging of the same beads continued in conventional mode resulting in movement and disintegration of the gold nanoparticles.
